# Patient Blood Management for Platelet Transfusion: Current Evidence, Safety Concerns and Practical Implementation in Hematology

**DOI:** 10.3390/jcm15218747

**Published:** 2026-11-09

**Authors:** Maria Eva Mingot-Castellano, Rocio Cardesa Cabrera, Maria Angeles Dominguez Muñoz, Maria Amparo Pimentel Villar, Nancy Rodriguez Torres

**Affiliations:** 1 Servicio de Hematología, Hospital Universitario Virgen del Rocio, Instituto de Biomedicina de Sevilla (IBIS/CSIC), Universidad de Sevilla, 41013 Sevilla, Spain; 2 Servicio de Hematología, Hospital de la Merced, Osuna, 41640 Sevilla, Spain; rocio.cardesa.sspa@juntadeandalucia.es; 3 Servicio de Hematología, Hospital Universitario Virgen de Valme. 41014 Sevilla, Spain; mariaa.dominguez.munoz.sspa@juntadeandalucia.es (M.A.D.M.); mariaa.pimentel.sspa@juntadeandalucia.es (M.A.P.V.); 4 Servicio de Hematología, Hospital Universitario Virgen del Rocio, 41013 Sevilla, Spain; nancy.rodriguez.torres.sspa@juntadeandalucia.es

**Keywords:** transfusion, platelets, hemovigilance, patient blood management, thrombocytopenia, thresholds, bleeding, hematologic malignancies, refractoriness, safety

## Abstract

Platelet transfusion is essential in hematology and oncology but remains characterized by practice variability, uneven certainty of evidence across clinical settings, short component shelf life and clinically relevant adverse effects. This narrative review integrates evidence on efficacy, thresholds and dosing, safety, and implementation of platelet-specific patient blood management (PBM). In stable non-bleeding therapy-induced thrombocytopenia, a restrictive prophylactic threshold of 10 × 10^9^/L is supported by randomized evidence and contemporary guidelines, whereas higher thresholds increase exposure without consistent clinical benefit. Therapeutic-only strategies reduce utilization but are not equally safe across populations, particularly in acute leukemia. Platelet PBM should therefore combine indication-specific protocols, a single-adult-dose policy with reassessment, audit and feedback, post-transfusion response monitoring, refractoriness management, inventory stewardship and multidisciplinary governance. The objective is not simply fewer transfusions but fewer unnecessary transfusions with preserved hemostatic safety.

## 1. Introduction and Rationale for Platelet-Specific PBM

Patient blood management (PBM) is a multidisciplinary, systematic, evidence-based and patient-centered approach intended to preserve endogenous hematopoiesis, minimize blood loss, and ensure rational, individualized use of blood components [1]. During the last two decades, PBM has become firmly established for red-cell transfusion through restrictive hemoglobin thresholds and proactive anemia management. Platelet-specific PBM (PBM-P), however, remains less mature and more heterogeneous [2,3,4,5]. This asymmetry largely reflects the evidence base: red-cell policy is supported by numerous large randomized trials, whereas platelet practice has historically depended on a smaller number of randomized studies focused mainly on prophylactic thresholds and dosing.

Implementation studies also show that education alone is unlikely to be sufficient. Sustainable change requires repeated measurement, feedback and clinical ownership. High-use services should receive their own utilization and appropriateness data, ideally alongside bleeding and adverse-event outcomes. A multidisciplinary transfusion committee can then identify whether excess use reflects knowledge gaps, order-set design, procedure pathways, delayed laboratory information or genuine case-mix differences. This governance structure is particularly important when introducing restrictive policies, because clinicians need a rapid mechanism for discussing exceptions and safety signals [5,6,7,8,9].

Hematology and oncology account for a substantial share of platelet support, much of it prophylactic; global blood-supply data provide important context, although they do not establish the proportion attributable to these specialties [10]. Intensive myelosuppressive chemotherapy and hematopoietic stem cell transplantation (HSCT) contribute to transfusion demand. Contemporary transplant guidance addresses blood-product selection to limit HLA immunization before HLA-mismatched allogeneic HSCT [11]. Post-transplantation cyclophosphamide (PTCy) is an important determinant of post-transplant transfusion requirements: Marco-Ayala et al. specifically assessed its impact in HLA-matched sibling peripheral blood stem cell transplantation, while Brissot et al. compared PTCy with anti-thymocyte globulin in a randomized trial of reduced-intensity allogeneic transplantation [12,13]. Advanced cellular therapies, particularly chimeric antigen receptor T-cell (CAR-T) therapy, may cause prolonged cytopenias, as recognized in EHA/EBMT consensus recommendations [14]. Real-world studies also document transfusion requirements after CAR-T infusion [15].

Despite evidence-based guidance, real-world platelet prescribing remains variable. Multicenter audits have documented substantial non-adherence and potentially inappropriate transfusion, creating a clear target for stewardship [3,16]. The 2025 AABB/ICTMG guideline provides a more restrictive contemporary framework and creates an opportunity to reassess legacy thresholds and routine practice [4]. Table 1 summarizes platelet transfusion thresholds and evidence across clinical scenarios, distinguishing guideline-based recommendations from settings requiring individualized decisions.

A central difficulty is that the clinical endpoint of platelet transfusion is not the platelet count itself but prevention or control of bleeding. The relationship between count and bleeding is nonlinear, and the same platelet count may represent very different clinical risks depending on the underlying disease, duration of thrombocytopenia, mucosal integrity, fever or sepsis, concomitant coagulation abnormalities, antithrombotic treatment and the anatomical consequences of a potential bleed. This makes platelet PBM intrinsically more dependent on clinical context than a simple laboratory-trigger model. It also explains why a policy that is appropriately restrictive in a stable patient with hypoproliferative thrombocytopenia may be inappropriate in active hemorrhage or in a patient undergoing a high-risk procedure. The practical challenge is therefore to standardize what can be standardized while preserving rapid individualized decision-making when risk is not captured by the platelet count alone [4,17,18].

Platelets are also a biologically and logistically fragile resource. Conventional storage at 20–24 °C under continuous agitation limits shelf life and favors bacterial proliferation, while unpredictable demand creates simultaneous risks of shortage and outdating. The term “platelet insecurity” describes the vulnerability of the supply chain to donor fluctuations, collection constraints, technical incidents and public health contingencies [19]. The financial impact is substantial because of collection, processing, testing, pathogen-reduction procedures, storage and hospital handling.

Platelet components differ in collection method, plasma versus platelet-additive-solution content, pathogen-reduction processing, and storage temperature and duration, subject to local regulatory requirements [20]. These characteristics may influence post-transfusion count increment (CI), corrected count increment (CCI), and inventory availability [20,21,22]. A lower increment alone should not automatically prompt an additional dose; decisions should also consider clinical bleeding, the achieved platelet count, the indication for transfusion, and patient-specific causes of poor response [23].

These logistic constraints have direct clinical implications. Unlike red-cell inventory, platelet stock cannot be expanded indefinitely to buffer fluctuations in demand. Hematology services may generate abrupt peaks in utilization during induction chemotherapy, transplantation or cellular-therapy programs, while emergency surgery and major hemorrhage compete for the same short-lived resource. PBM-P, therefore, has a dual objective: reducing patient exposure to transfusions that are unlikely to provide benefit and improving the resilience of the platelet supply for patients in whom transfusion is genuinely necessary. In this sense, clinical appropriateness and inventory stewardship are complementary rather than competing goals [19].

Finally, platelet transfusion is not a benign intervention. Acute febrile and allergic reactions, bacterial contamination and sepsis, transfusion-associated circulatory overload (TACO), transfusion-related acute lung injury (TRALI), HLA/HPA alloimmunization and platelet refractoriness are clinically relevant complications [24,25,26,27]. PBM-P should therefore be understood not simply as “transfusing less” but as a safety and stewardship framework: transfuse when expected benefit exceeds risk, use the minimum effective dose, reassess response, investigate ineffective support and preserve access to an essential but scarce component.

## 2. Clinical Effectiveness: Prophylactic Versus Therapeutic Platelet Transfusion

Prophylactic platelet transfusion is administered to prevent bleeding in thrombocytopenic patients without active hemorrhage, whereas therapeutic transfusion is reserved for clinically relevant bleeding or an urgent hemostatic requirement. The best-studied setting is therapy-induced hypoproliferative thrombocytopenia in hematologic malignancy and HSCT [28,29,30,31].

In stable non-bleeding patients with therapy-induced hypoproliferative thrombocytopenia, randomized trials and systematic reviews comparing prophylactic thresholds of 10 × 10^9^/L with 20–30 × 10^9^/L support the lower threshold as a strategy that reduces platelet exposure without a demonstrated increase in major bleeding or mortality [29,32,33]. These findings underpin the contemporary recommendation to use 10 × 10^9^/L as the standard prophylactic trigger in this setting [4].

The importance of this evidence is not merely numerical. Historically, higher thresholds were often adopted in an attempt to create a wider safety margin, particularly during intensive chemotherapy. However, increasing the prophylactic trigger predictably increases the number of transfusion episodes and donor exposures, while randomized comparisons have not shown a corresponding improvement in the outcomes that matter most to patients. The PBM interpretation is therefore that a higher platelet count after transfusion should not be confused with greater clinical effectiveness. The relevant balance is between bleeding prevention and the burden and risk created by additional transfusion exposure [29,32,33].

The comparison between routine prophylaxis and a therapeutic-only strategy is more complex. Wandt et al. showed that a therapeutic strategy substantially reduced platelet transfusions, but severe bleeding occurred more frequently in acute myeloid leukemia, including intracranial hemorrhage [28]. In TOPPS, omission of prophylactic transfusion did not meet non-inferiority criteria: WHO grade 2–4 bleeding occurred in 50% of patients without prophylaxis compared with 43% receiving prophylaxis [34]. Meta-analytic evidence therefore supports an important distinction: therapeutic-only strategies reduce component exposure but at the cost of more clinically significant bleeding when applied indiscriminately [30].

The WANDT and TOPPS studies also illustrate why platelet PBM should not be equated with eliminating prophylaxis. Both studies achieved meaningful reductions in platelet utilization when prophylactic transfusions were withheld, but the bleeding signal differed according to the population studied. In acute leukemia, where thrombocytopenia is often prolonged and coexists with infection, mucosal injury and intensive treatment, the increased risk of severe hemorrhage makes a universal therapeutic-only strategy difficult to justify [28]. In selected clinically stable autologous HSCT recipients, the shorter duration of thrombocytopenia may allow a therapeutic-only approach under close surveillance and with immediate access to platelet support [4,28,34]. This should not be extrapolated to acute leukemia or allogeneic HSCT.

Platelet count alone is an imperfect predictor of hemorrhage. Prospective analyses demonstrate that fever, infection, anemia, mucosal damage, coagulopathy, medications, prior bleeding and the duration and trajectory of thrombocytopenia modify bleeding risk [17,18]. PBM therefore requires numerical thresholds to be interpreted within the whole bleeding-risk phenotype rather than used as isolated laboratory triggers. Severe anemia may impair platelet margination toward the vessel wall and thereby worsen primary hemostasis in thrombocytopenia. Correction of clinically significant anemia should be assessed within an individualized red-cell PBM strategy; this does not justify transfusing red cells solely to reach an arbitrary hematocrit target [35].

This distinction also affects how bleeding outcomes should be interpreted in trials and audits. WHO grade ≥2 bleeding is frequently used as an endpoint, but it encompasses events of different clinical relevance, whereas rare critical-site hemorrhage is difficult to study because adequately powered trials would require very large sample sizes. Furthermore, transfusion may be administered in response to early bleeding, making exposure itself partly a marker of worsening clinical status. These methodological issues explain why evidence is strongest for common prophylactic scenarios and much weaker for critical illness, procedures and complex coagulopathies. They also support monitoring both utilization and patient-centered safety outcomes in PBM programs rather than using transfusion reduction as a surrogate for benefit [17,18,30].

Clinical bleeding assessment is essential but difficult to standardize. WHO bleeding grades and the Bleeding Severity Measurement Scale (BSMS) use different criteria; their definitions, assessment methods and validation populations should be considered before comparing results across studies [36,37,38]. Red-cell transfusion requirements and estimated or measured blood loss may provide useful adjunct information, but transfusion use also reflects baseline anemia and transfusion practice, while perioperative blood-loss estimates depend on the procedure and measurement method [39,40].

Evidence in the intensive care unit is substantially weaker. Prospective observational studies demonstrate wide variation in indications and frequent poor post-transfusion increments, particularly in sepsis and hematologic malignancy [41,42]. A propensity score analysis suggested that a more restrictive strategy could reduce platelet use without an observed increase in grade ≥2 bleeding or 28-day mortality [43]. Observational associations between platelet transfusion and mortality or thrombosis in sepsis and consumptive disorders remain vulnerable to confounding by indication and should not be interpreted as proof of transfusion-related harm [44,45].

The ICU is a particularly important example of the limits of count-based practice. Thrombocytopenia in critical illness is often multifactorial and may reflect sepsis, disseminated consumption, organ failure, extracorporeal support, medications or marrow suppression. Consequently, a poor post-transfusion increment does not necessarily indicate immune refractoriness, and repeated empirical dosing may add exposure without achieving a durable platelet response [41,42]. A PBM approach in this setting should first clarify the indication—active bleeding, procedure or prophylaxis—and then consider the likelihood that transfusion will produce a clinically useful hemostatic effect.

## 3. Indications, Thresholds and Dose: Consensus and Remaining Uncertainty

The area of greatest international consensus is stable, non-bleeding central thrombocytopenia during chemotherapy or allogeneic HSCT: the 2025 AABB/ICTMG guideline recommends prophylactic transfusion at <10 × 10^9^/L [4]. The guideline, developed after systematic review using GRADE methodology, found no demonstrated increase in mortality or severe bleeding with restrictive practice in the populations evaluated [4,46]. Older AABB and British guidance broadly support the same direction, although recommendations diverge in selected procedural and high-risk situations [31,47].

Procedural thresholds remain a major source of variability. AABB/ICTMG 2025 recommends or conditionally suggests restrictive thresholds that vary by procedure, including <10 × 10^9^/L for compressible-site central venous catheter placement, <20 × 10^9^/L before lumbar puncture or low-risk interventional radiology, and <50 × 10^9^/L for high-risk interventional radiology or major non-neuraxial surgery [4]. The certainty of evidence is lower than in stable hypoproliferative thrombocytopenia, and these values should be interpreted as decision boundaries rather than automatic targets. These values should not be treated as mandatory targets. Procedure type, ability to achieve local hemostasis, operator experience, platelet trend and function, coagulopathy, antithrombotic therapy and urgency all modify risk.

This is clinically relevant because procedural thresholds are frequently transformed in practice from conditional recommendations into fixed pre-procedure targets. Such an approach may generate repeated transfusions to reach an arbitrary count even when the bleeding site is compressible, ultrasound-guided or otherwise associated with low bleeding risk. Conversely, a numerically acceptable count should not provide false reassurance for a procedure in a patient with additional hemostatic abnormalities. The PBM objective is therefore not to replace one universal threshold with another but to embed procedure-specific guidance into a broader assessment of technical and patient-related bleeding risk [4,47].

Complex contemporary settings remain evidence gaps rather than areas for firm platelet-specific recommendations. In CAR-T recipients, practice is largely extrapolated from general oncohematology because no consolidated platelet-specific threshold has been established. Thrombocytopenia may coexist with infection, inflammation, coagulopathy, endothelial activation and anticoagulation. In cancer-associated thrombosis with thrombocytopenia, consensus and cohort data generally support full-dose anticoagulation when platelet counts are ≥50 × 10^9^/L without routine transfusion support, whereas lower counts require individualized strategies based on thrombotic acuity and bleeding risk [48,49].

Dose is a second major PBM lever. When transfusion is indicated, one adult apheresis dose or equivalent should generally be administered, followed by clinical reassessment before repetition [4,31]. The PLADO trial demonstrated that higher prophylactic doses did not reduce bleeding; lower doses reduced the total quantity of platelets transfused but required more frequent transfusion episodes [50]. These data support a “single adult dose, then reassess” policy and argue against routine initial multi-unit orders in non-bleeding patients.

The dosing evidence is particularly suitable for implementation because it can be translated directly into prescribing behavior. A default order for one adult therapeutic dose reduces the tendency to prescribe two units pre-emptively and creates a deliberate reassessment point. That reassessment may be clinical in active bleeding and may include a post-transfusion platelet count when the objective is prophylaxis, preparation for a procedure or evaluation of suspected refractoriness. This approach does not imply that a second dose is never appropriate; rather, it requires the expected benefit of additional platelets to be reconsidered after the response to the first dose is known [4,50].

The practical implication is that platelet thresholds should function as decision-support boundaries rather than isolated triggers. A request should identify the indication, latest platelet count and sampling time, bleeding status and site, planned procedure and timing, and relevant modifiers. Exceptions are expected in complex medicine, but they should be explicit, clinically justified and auditable.

## 4. Safety, Adverse Events and Cumulative Exposure

Any platelet PBM program must incorporate safety as a primary outcome. The objective is not merely to reduce utilization but to reduce avoidable exposure while preserving hemostatic safety. Each transfusion introduces biological material, volume and donor antigens, and cumulative exposure becomes particularly relevant when support is required over weeks or months [1,4,46].

Febrile non-hemolytic and allergic reactions are among the most frequent acute complications. Bacterial contamination is a distinctive platelet safety concern because room-temperature storage facilitates bacterial proliferation. Detection and pathogen-reduction strategies have reduced but not eliminated this risk [24,25,26]. Cardiopulmonary complications include TACO, particularly in older patients and those with cardiac or renal dysfunction, and TRALI, characterized by acute hypoxemia and non-cardiogenic pulmonary edema [27,51]. Transfusion-associated circulatory overload (TACO) can occur after platelet transfusion, including when platelets are administered with other blood components or intravenous fluids. Risk is influenced by the patient’s fluid balance, transfusion volume and infusion rate, as well as recipient susceptibility—particularly cardiac or renal dysfunction [52]. Finally, patients with underlying marrow failure or immune dysregulation, including severe aplastic anemia and myelodysplastic syndromes, may have substantial cumulative transfusion exposure and should be evaluated carefully for alloimmunization; whether disease-specific susceptibility is independent of exposure requires supporting evidence [4,48,49].

Repeated platelet transfusion can lead to HLA/HPA alloimmunization and refractoriness. Most poor increments are non-immune and associated with infection, fever, active bleeding, disseminated intravascular coagulation, splenomegaly or drugs; immune causes are driven mainly by HLA antibodies [25,53]. Persistent inadequate increments should trigger investigation rather than automatic additional units. A platelet count approximately 10–60 min after transfusion, with corrected count increment when appropriate, can guide assessment [23].

Refractoriness is particularly relevant to PBM because it can generate a self-perpetuating cycle of ineffective transfusion. When an inadequate increment is followed automatically by another unselected platelet dose, utilization rises while the underlying mechanism remains unresolved. Early distinction between non-immune consumption and immune refractoriness is therefore both a clinical and stewardship intervention. In a patient with ongoing bleeding, sepsis, fever or disseminated coagulation, correction of the underlying driver may be more important than repeated attempts to normalize the platelet count. When immune refractoriness is suspected, coordinated investigation with the Blood Bank allows scarce crossmatch-compatible or HLA-selected components to be reserved for patients most likely to benefit [23,53].

Both the uncorrected count increment (CI) and corrected count increment (CCI) are surrogate measures of post-transfusion platelet recovery rather than direct measures of clinical hemostatic efficacy. Timing of sampling, component platelet content and storage characteristics, body surface area, splenic sequestration, fever, bleeding and consumption may alter their interpretation. A satisfactory increment does not guarantee bleeding control, while a low increment does not by itself establish immune refractoriness [54].

ABO-identical or compatible platelets generally provide better post-transfusion increments; however, the 2026 systematic review found very-low-certainty evidence for differences in clinically significant bleeding, mortality, acute transfusion reactions or refractoriness [55]. Thus, ABO-identical/compatible components should be prioritized when feasible, particularly in repeatedly transfused patients, without compromising timely access to platelet support. Transfusion-related immunomodulation (TRIM) describes biologically plausible immunosuppressive and pro-inflammatory effects mediated by cellular and soluble factors [56]. Observational associations between platelet transfusion and thrombosis or mortality should be interpreted cautiously because confounding by indication is substantial [45,57].

The corrected count increment should likewise be used selectively rather than as a routine endpoint after every transfusion. Its greatest value is in patients with repeated inadequate responses, where it helps establish whether the observed increment is disproportionate to the platelet dose and recipient size. The clinical response remains essential: an apparently satisfactory laboratory increment does not replace assessment of hemostasis in a bleeding patient, and a low increment in a patient with major ongoing consumption may not justify an immune work-up. PBM therefore links laboratory monitoring to a specific clinical question rather than increasing testing indiscriminately [23].

Patients with leukemia, HSCT and CAR-T therapy are particularly exposed because platelet support may continue for prolonged periods. In the DESCAR-T cohort, more than half of patients with large B-cell lymphoma treated with CAR-T required transfusion support during the six months after infusion, with the greatest utilization during the first month [15]. Minimizing exposure in these settings means avoiding transfusions without expected benefit, not withholding clinically indicated support.

## 5. Platelet PBM Strategies, Implementation and Future Perspectives

Platelet PBM should become an institutional stewardship program that defines when to transfuse, how much to transfuse, how response is assessed and how safety is monitored. The cornerstone is an indication-specific policy separating non-bleeding prophylaxis, planned procedures, active bleeding and specialist-directed exceptions. Platelet count alone should not trigger transfusion; bleeding phenotype, platelet trend and function, procedure-related risk, coagulopathy, infection, antithrombotic therapy and underlying disease must also be considered [4]. The principal components of a practical platelet PBM implementation bundle, together with suggested quality indicators, are summarized in Table 2.

Electronic orders should require the indication, latest platelet count and sampling time, bleeding status/site, planned procedure and timing, and justification for exceptions or repeated doses. Decision support can display the relevant threshold, flag prophylactic orders above it and discourage initial multi-unit orders while preserving immediate override for major bleeding. Audit should be coupled with targeted feedback and multidisciplinary governance. These principles can be integrated into a stepwise clinical decision-support pathway, as proposed in Figure 1.

The electronic request is an especially powerful implementation point because it occurs before exposure has taken place. Mandatory documentation can make the indication visible, while context-sensitive decision support can display the relevant threshold and identify orders that fall outside the local pathway. The aim is not to create a rigid electronic barrier: major bleeding must always allow immediate override. Instead, the system should make the default action evidence-based and require active justification when clinicians depart from that default. The same data fields can subsequently support audit, allowing services to distinguish appropriate exceptions from systematic overuse.

Evidence from implementation studies suggests that educational interventions, when used in isolation, rarely achieve sustained improvements in transfusion practice. Long-term effectiveness depends on continuous monitoring, regular performance feedback, and active engagement of clinical teams. Departments with high transfusion activity should have access to service-specific data on utilization patterns and adherence to appropriate transfusion criteria, complemented whenever possible by information on bleeding complications and transfusion-related adverse events. Within this framework, multidisciplinary transfusion committees play a central role in identifying the underlying drivers of potentially inappropriate use, including insufficient awareness of recommendations, limitations in electronic ordering systems, established procedural practices, delays in laboratory reporting, or differences in patient complexity. Such oversight becomes especially relevant when implementing restrictive transfusion strategies, as it provides a structured process for evaluating clinical exceptions, addressing safety concerns, and adapting practice based on emerging evidence and local outcomes [5,6,7,8,9].

Post-transfusion assessment is another underused PBM intervention. Repeated transfusion without an adequate increment should trigger investigation. Non-immune causes should be assessed first; persistent inadequate increments should prompt HLA/HPA antibody testing and early Blood Bank involvement, with crossmatch-compatible or HLA-selected platelets when immune refractoriness is demonstrated [23,53].

Inventory stewardship is integral because short shelf life and unpredictable demand create simultaneous risks of shortage and wastage. Demand forecasting, timely release of unused reservations, first-expire-first-out allocation and real-time monitoring of outdating should be incorporated into Blood Bank practice. Modeling reduced wastage from 10.1% to approximately 5% and shortages from 6.5% to 1.7–2.1% [58].

Inventory metrics should therefore be included in the PBM dashboard rather than treated as a separate Blood Bank problem. Useful measures include outdating, emergency shortages, redistribution, the proportion of reserved components released unused and demand by clinical service. Linking anticipated chemotherapy, HSCT, CAR-T and procedural activity to stock planning may improve forecasting, while real-time review of expiry allows redistribution before components are lost. The modeling data are encouraging but should be interpreted as proof of logistic potential rather than evidence of improved clinical outcomes [58].

Cold-stored platelets can extend product availability and retain favorable immediate hemostatic properties, making them particularly attractive for actively bleeding patients; however, processing variability and shorter in vivo survival remain important limitations when extrapolating their use to prophylactic support in hematology [59]. Clinical evidence for cold-stored platelets should be interpreted according to indication. In patients with active bleeding during cardiac surgery, the CHIPS randomized trial found platelets stored cold for up to 21 days noninferior to room-temperature platelets on a prespecified hemostatic efficacy score; it did not establish superiority. Trauma trials have assessed early cold-stored platelet transfusion, but their results do not establish improved survival or long-term functional outcome. These findings should not be extrapolated to prophylactic support during prolonged hypoproliferative thrombocytopenia, for which clinical benefit remains uncertain [60]. Pathogen-reduction technologies improve microbiological safety, but efficacy should be considered technology-specific: a 2024 meta-analysis found no overall increase in severe bleeding with pathogen-reduced platelets, while clinically significant bleeding differed between amotosalen/UVA- and riboflavin/UV-treated components [61]. Cryopreservation offers major logistic advantages and prolonged storage, but the CLIP-II randomized trial in cardiac surgery did not establish noninferiority of cryopreserved platelets to conventional liquid-stored platelets for 24 h bleeding control and found greater blood loss and use of other blood components in several secondary outcomes [62]. These technologies should therefore be viewed as context-specific tools rather than interchangeable replacements for conventional platelets.

TPO-RAs may complement platelet PBM in persistent thrombocytopenias but are not substitutes for transfusion in acute bleeding because their effect is delayed The uses described below are off-label, although an increasing body of evidence is emerging to support their potential role in selected clinical settings. Chemotherapy-induced thrombocytopenia (CIT) has the strongest emerging evidence. NCCN recommends considering romiplostim for CIT (category 2A) to maintain chemotherapy dose schedule and intensity when expected benefit outweighs risk [63]. In the phase 3 RECITE trial, 165 patients with persistent CIT with gastrointestinal cancers receiving oxaliplatin-based chemotherapy were randomized to romiplostim or placebo. No CIT-induced chemotherapy dose modification during cycles 2 and 3 occurred in 84% versus 36% (OR 10.16; 95% CI, 4.44–23.72; *p* < 0.001) [64]. Thromboembolic events occurred in 2% versus 0%. Earlier romiplostim data [65] and ISTH guidance supporting TPO-RA consideration when inadequate platelet recovery would otherwise require chemotherapy delay or dose reduction [66]. In a phase III trial involving patients with ovarian, bladder or lung cancer and chemotherapy-induced thrombocytopenia, avatrombopag did not improve the composite primary endpoint compared with placebo [67]. Subsequently, the randomized, placebo-controlled phase II ACT-GI trial found that avatrombopag improved a composite of platelet recovery and chemotherapy delivery in patients with gastrointestinal cancers and persistent chemotherapy-induced thrombocytopenia: 70% versus 17% met the endpoint. The trials differed in population, dosing and endpoint definition; their results should not be extrapolated uncritically across TPO-RAs or clinical settings [68].

Evidence after cellular therapy and HCT is less robust. In a Spanish multicenter cohort of 38 patients with platelet-transfusion-dependent thrombocytopenia after anti-CD19 CAR-T therapy, eltrombopag was associated with transfusion independence in 29 (76.3%); the uncontrolled design prevents causal attribution [69]. After HCT, eltrombopag is the most studied TPO-RA. A review identified 64 reports including 1730 patients but only five randomized trials, supporting selective treatment of established prolonged thrombocytopenia rather than routine early administration to accelerate engraftment [70].

PBM endpoints differ by setting: preservation of chemotherapy relative dose intensity is central in CIT, whereas independence may be more appropriate after CAR-T or HCT. TPO-RAs should be used in diagnosis-specific pathways and never delay transfusion when immediate hemostatic support is required. Avatrombopag reduces peri-procedural transfusion or rescue therapy in adults with chronic liver disease [71], whereas prophylactic tranexamic acid did not reduce clinically relevant bleeding in thrombocytopenic hematology patients [72].

The future of platelet PBM should be individualized, data-driven and auditable. The objective is not the lowest possible platelet consumption but the lowest avoidable transfusion exposure compatible with timely hemostatic support and demonstrable patient safety.

The evidence hierarchy used in this review is as follows: (1) guideline-supported recommendations with randomized evidence, particularly the 10 × 10^9^/L prophylactic threshold in stable hypoproliferative thrombocytopenia; (2) conditional recommendations with lower-certainty evidence, particularly many procedure-related thresholds; and (3) evidence gaps requiring individualized practice, including CAR-T–associated thrombocytopenia, several ICU scenarios, and thrombocytopenia during anticoagulation.

## 6. Conclusions

The strongest evidence supports restrictive prophylaxis at 10 × 10^9^/L in stable thrombocytopenia, avoidance of routinely higher thresholds, and one adult dose followed by reassessment. Therapeutic-only strategies may be appropriate in selected populations but cannot be generalized to acute leukemia or other high-risk settings. Procedural thresholds, critical illness, CAR-T therapy, anticoagulation and individualized prediction of bleeding remain important evidence gaps.

A mature platelet PBM program should combine evidence-based thresholds with individualized bleeding-risk assessment, active stewardship, post-transfusion response monitoring, prevention and management of refractoriness, inventory optimization and surveillance of both bleeding and transfusion-related harm. The central objective is not simply fewer transfusions but fewer unnecessary transfusions with preserved or improved patient safety.

The next phase of platelet PBM should focus on prospective implementation research. Future studies should report not only units avoided but also clinically significant and critical-site bleeding, mortality, procedure delays, transfusion reactions, refractoriness, patient exposure to donors, wastage and cost. Better risk-prediction tools may ultimately allow prophylaxis to be targeted according to bleeding phenotype rather than platelet count alone, but such tools require validation before they can replace current evidence-based thresholds. Until then, the most defensible strategy is a layered model: restrictive defaults where evidence is strong, individualized decisions where evidence is conditional, and explicit recognition of evidence gaps rather than extrapolation presented as certainty.

## Figures and Tables

**Figure 1:**
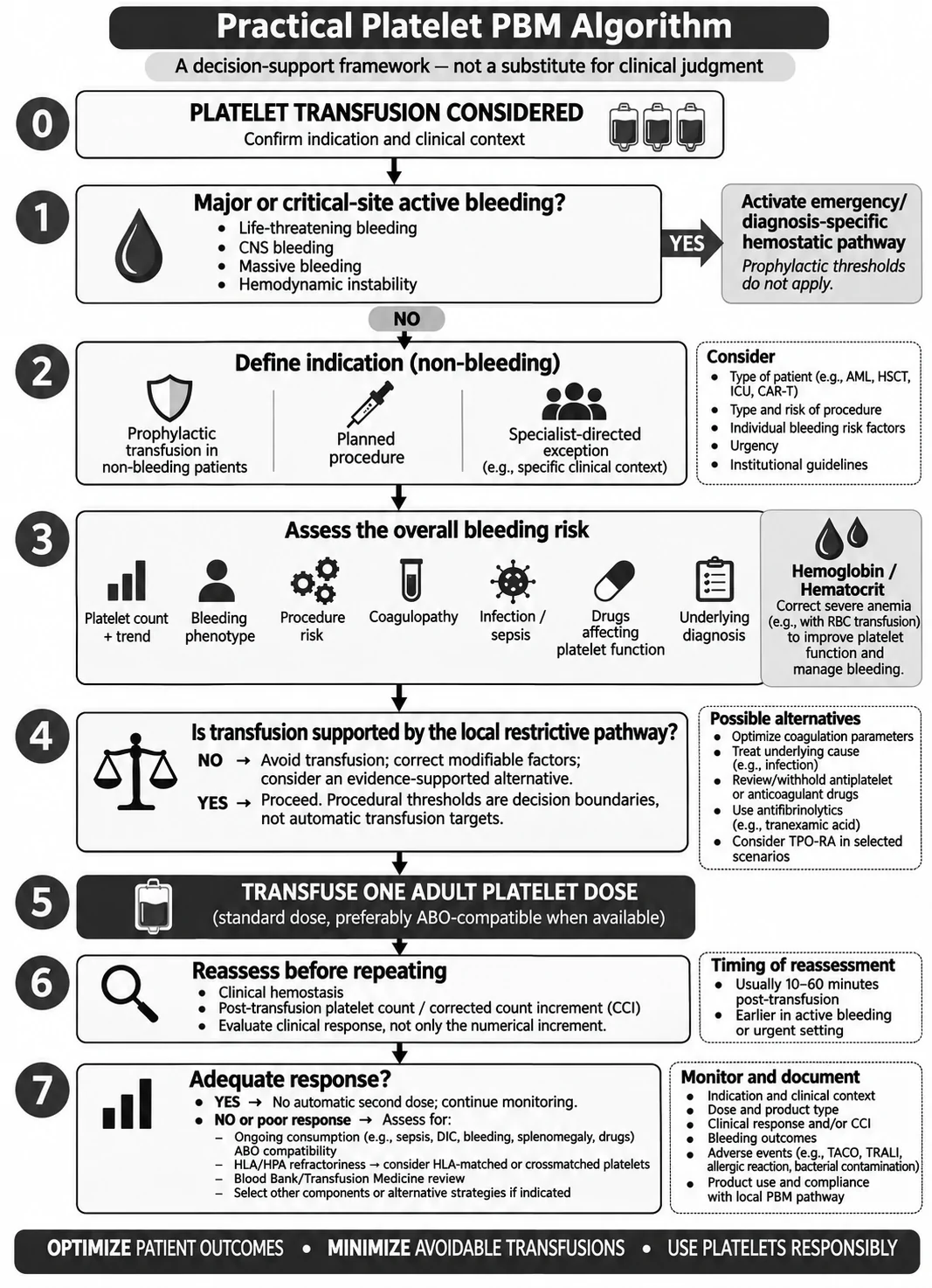
Proposed practical platelet PBM algorithm. The algorithm is intended as a PBM decision-support framework. Procedural thresholds should not be interpreted as mandatory targets, and major or critical-site bleeding requires diagnosis-specific emergency management. CCI, corrected count increment; HLA, human leukocyte antigen; HPA, human platelet antigen. Figure 1 has been prepared with assistance from OpenAI ChatGPT (GPT-6; accessed October 2026) for language editing.

**Table 1:** Practical platelet transfusion thresholds within a PBM framework.

Clinical Scenario	Threshold/Strategy	Evidence/Consensus	PBM-Based Approach
Stable non-bleeding hypoproliferative thrombocytopenia	<10 × 10^9^/L	Guideline-supported; randomized evidence [SOURCE TO VERIFY: guideline/trial reference]	Avoid routinely higher thresholds
Allogeneic HSCT, non-bleeding	<10 × 10^9^/L	Guideline-supported [SOURCE TO VERIFY: guideline/trial reference]	Integrate clinical bleeding risk
Selected stable autologous HSCT	Therapeutic-only may be considered	Selected population; trial/subgroup evidence [SOURCE TO VERIFY: guideline/trial reference]	Close surveillance required
Lumbar puncture	<20 × 10^9^/L	Guideline-supported; lower-certainty evidence [SOURCE TO VERIFY: guideline/trial reference]	Not an automatic target
Compressible-site CVC	<10 × 10^9^/L	Conditional; low/very low certainty [SOURCE TO VERIFY: guideline/trial reference]	Consider local hemostasis
Low-risk interventional radiology	<20 × 10^9^/L	Conditional; low/very low certainty [SOURCE TO VERIFY: guideline/trial reference]	Individualize
High-risk IR/major non-neuraxial surgery	<50 × 10^9^/L	Conditional; low/very low certainty [SOURCE TO VERIFY: guideline/trial reference]	Consider coagulopathy/antithrombotics
Major/critical-site active bleeding	No prophylactic threshold	Clinical emergency pathway [SOURCE TO VERIFY: guideline/trial reference]	Treat bleeding and hemostatic defect
CAR-T–associated thrombocytopenia	No dedicated universal threshold	Evidence gap [SOURCE TO VERIFY: guideline/trial reference]	Extrapolate cautiously from oncohematology; individualize
ICU thrombocytopenia without active bleeding	No universal platelet-specific threshold	Evidence gap/observational data [SOURCE TO VERIFY: guideline/trial reference]	Avoid automatic count-based transfusion
Cancer-associated thrombosis with thrombocytopenia	No universal platelet transfusion target	Evidence gap [SOURCE TO VERIFY: guideline/trial reference]	Balance thrombotic and bleeding risk; coordinate anticoagulation
Non-thrombocytopenic intracranial hemorrhage/cardiovascular surgery	Separate recommendations, if applicable; not interchangeable with prophylactic thrombocytopenia thresholds	[VERIFY AABB/ICTMG 2025 exact wording, evidence and applicability]	Assess indication-specific guidance and avoid extrapolation

**Table 2:** Practical platelet PBM implementation bundle.

PBM Domain	Practical Intervention	Suggested Indicator
Indication	Indication-specific protocol	% meeting predefined indication
Threshold	Restrictive threshold in ordering	% prophylactic transfusions above threshold
Dose	One adult dose; reassess	% initial multi-unit orders
Decision support	Mandatory indication/count/bleeding/procedure	Override rate
Audit/feedback	Service-level review	Appropriate transfusion rate
Safety	Monitor bleeding, mortality, reactions	Bleeding/adverse-event rates
Response	Post-transfusion increment/CCI when appropriate	% low increments investigated
Refractoriness	Non-immune causes → HLA/HPA work-up	Ineffective transfusions before review
Inventory	Forecasting and expiry surveillance	Wastage/shortage rates
Governance	Multidisciplinary committee	Regular PBM dashboard

## Data Availability

No new data were created or analyzed in this study. Data sharing is not applicable to this article.
